# PDGF-BB signaling *via* PDGFR-β regulates the maturation of blood vessels generated upon vasculogenic differentiation of dental pulp stem cells

**DOI:** 10.3389/fcell.2022.977725

**Published:** 2022-10-19

**Authors:** Zhaocheng Zhang, Kristy A. Warner, Andrea Mantesso, Jacques E. Nör

**Affiliations:** ^1^ Angiogenesis Research Laboratory, Department of Cariology, Restorative Sciences and Endodontics, University of Michigan School of Dentistry, Ann Arbor, MI, United States; ^2^ Department of Biomedical Engineering, University of Michigan College of Engineering, Ann Arbor, MI, United States; ^3^ Department of Otolaryngology, University of Michigan School of Medicine, Ann Arbor, MI, United States

**Keywords:** smooth muscle cells, pericytes, self-renewal, angiogenesis, tissue regeneration, vasculogenesis

## Abstract

A functional vascular network requires that blood vessels are invested by mural cells. We have shown that dental pulp stem cells (DPSC) can undergo vasculogenic differentiation, and that the resulting vessels anastomize with the host vasculature and become functional (blood carrying) vessels. However, the mechanisms underlying the maturation of DPSC-derived blood vessels remains unclear. Here, we performed a series of studies to understand the process of mural cell investment of blood vessels generated upon vasculogenic differentiation of dental pulp stem cells. Primary human DPSC were co-cultured with primary human umbilical artery smooth muscle cells (HUASMC) in 3D gels in presence of vasculogenic differentiation medium. We observed DPSC capillary sprout formation and SMC recruitment, alignment and remodeling that resulted in complex vascular networks. While HUASMC enhanced the number of capillary sprouts and stabilized the capillary network when co-cultured with DPSC, HUASMC by themselves were unable to form capillary sprouts. *In vivo*, GFP transduced human DPSC seeded in biodegradable scaffolds and transplanted into immunodeficient mice generated functional human blood vessels invested with murine smooth muscle actin (SMA)-positive, GFP-negative cells. Inhibition of PDGFR-β signaling prevented the SMC investment of DPSC-derived capillary sprouts *in vitro* and of DPSC-derived blood vessels *in vivo*. In contrast, inhibition of Tie-2 signaling did not have a significant effect on the SMC recruitment in DPSC-derived vascular structures. Collectively, these results demonstrate that PDGF-BB signaling *via* PDGFR-β regulates the process of maturation (mural investment) of blood vessels generated upon vasculogenic differentiation of human dental pulp stem cells.

## Introduction

Vasculogenesis refers to the *de novo* formation of blood vessels from endothelial progenitors, which assemble into a primary capillary plexus during early embryonic development (Risau, 1997). The sprouting, branching and intussusceptive growth from pre-existing capillaries is named angiogenesis and is followed by subsequent stabilization of these vessels by mural cells (Carmeliet, 2000, Carmeliet, 2003). As such, a functional vascular network requires the nascent vessels to mature into more stable blood vessels. The association of pericytes and smooth muscle cells (SMC) with newly formed vessels regulates endothelial cell proliferation, survival, migration, vascular branching, blood flow, and vascular permeability (Jain, 2003). While the process of vessel maturation during early development is well understood, the mechanisms underlying the maturation of blood vessels generated *via* vasculogenic differentiation of postnatal mesenchymal stem cells remain largely unclear. Such knowledge is critical to optimize the use of these stem cells in tissue regeneration/engineering.

Platelet-derived growth factor (PDGF)-BB, a chemoattractant for SMCs in physiological conditions (Lindahl et al., 1998) has a critical role in the stabilization of nascent blood vessels *via* signaling through its receptor PDGFR-β. Pericytes and SMCs express PDGFR-β and smooth muscle actin-α (SMA-α) (Smyth et al., 2018). PDGF-BB signaling promotes vascular network maturation through the recruitment and expansion of mural cells (SMCs and pericytes) (Lindblom et al., 2003). PDGFR-β+ progenitor perivascular cells (PPCs) can differentiate into pericytes that regulate vessel stability and vascular survival in tumors, inhibition of PDGFR-β signaling eliminated PPCs and mature pericytes around tumor vessels leading to vascular hyperdilation and endothelial cell apoptosis (Song et al., 2005). Insufficient recruitment of mural cells results in endothelial cells (ECs) fragility, vessel enlargement, bleeding, impaired perfusion, and hypoxia in embryos lacking PDGF-BB (Hellström et al., 2001). Further, deletion of PDGF-BB results in embryonic lethality as endothelial cells appear to be unable to attract PDGFR-β-positive pericyte progenitor cells, resulting in leaky and hemorrhagic phenotypes (Lindahl et al., 1997). Mural cells stabilize nascent vessels by inhibiting endothelial proliferation and migration, and by stimulating production of extracellular matrix. They thereby provide hemostatic control and protect new endothelium-lined vessels against rupture or regression (Benjamin et al., 1998; Carmeliet, 2000).

Stem cells are characterized by multipotency and self-renewal and play critical roles in embryonic development and tissue regeneration. Mesenchymal stem cells (MSC) derived from the neural crest are essential for tooth development. Dental pulp stem cells (DPSC) from permanent teeth (Gronthos et al., 2000) and stem cells from human exfoliated deciduous teeth (SHED) (Miura et al., 2003) display multipotency, i.e., have the ability to differentiate into osteoblasts, odontoblasts, adipocytes, and neuronal cells (Miura et al., 2003; Huang et al., 2009; Feng et al., 2011; Wang et al., 2012; Saito et al., 2015), and also exhibit self-renewal capacity (Cucco et al., 2020; Oh et al., 2020). DPSC cells are considered to have potential utility in the treatment of several systemic diseases such as spinal cord injuries, myocardial infarction, diabetes, liver diseases, and immune diseases. In addition, they have demonstrable utility in regenerative medicine approaches. Notably, when compared with bone marrow derived mesenchymal stem cells, it appears that DPSCs might have higher regenerative potential (Yamada et al., 2019). Using DPSC and SHED as models of postnatal mesenchymal stem cells, we demonstrated that these cells undergo vascular endothelial differentiation to form capillary-like structures *in vitro* and functional blood vessels when seeded in biodegradable scaffolds and transplanted into murine hosts (Bento et al., 2013; Sakai et al., 2010; Sasaki et al., 2020; Zhang et al., 2016; Zhang et al.,2021). Here, we demonstrated the function of PDGF-BB signaling through PDGFR-β in the maturation of blood vessels generated through vasculogenic differentiation of dental pulp mesenchymal stem cells.

## Materials and methods

### Cell culture

Dental pulp stem cells (DPSC; Lonza, Walkersville, MD) and stem cells from human exfoliated deciduous teeth (SHED, kindly provided by Songtao Shi, University of Pennsylvania) were cultured in α-Minimum Essential Medium (α-MEM; Invitrogen, Carlsbad, CA, United States) supplemented with 5%–20% fetal bovine serum (FBS, Invitrogen) and 1% penicillin/streptomycin (Invitrogen) at 37°C and 5% CO_2_. Human umbilical artery smooth muscle cells (HUASMC, ScienCell Research Laboratories; Carlsbad, CA) were cultured in smooth muscle cell (SMC) medium with cell growth supplement (ScienCell Research Laboratories). Human dermal microvascular endothelial cells (HDMEC; Lonza, Walkersville, MD) were cultured in endothelial growth medium-2 for microvascular cells (EGM2-MV; Lonza). Cells were cultured with 0–20 ng/ml recombinant human PDGF-BB and/or bFGF (R&D Systems) in presence of 0–10 µM SB-203580 (Tie-2 kinase inhibitor; Selleckchem, Houston, TX) or 0–25 nM Ki11502 (PDGFR inhibitor; Millipore/Sigma, St. Louis, MO) for several time points.

### Western blots

Whole cell lysates were prepared with 1% Nonidet P-40 (NP-40) lysis buffer (50 mM Tris-HCL, PH 7.4, 10% glycerol, 200 mM NaCl and 2 mM MgCl2) containing protease inhibitors. Protein samples were loaded onto 8%–15% SDS-PAGE. Membranes were blocked with 5% non-fat milk in 1X TBS wash buffer containing 0.3% Tween-20, then incubated with the following primary antibodies overnight at 4°C: rabbit anti-human VEGFR2, mouse anti-human CD31 (Santa Cruz Biotechnology, Santa Cruz, CA, United States); rabbit anti-human Bmi-1, PDGFR-α, phosphor-PDGFR-β, PDGFR-β, phosphor-Tie-2, Tie-2, phosphor-AKT, AKT (Cell Signaling, Danvers, MA, United States); mouse anti-human smooth muscle actin-α (SMA-α), mouse anti-GAPDH (Millipore/Sigma). Affinity-purified secondary antibodies conjugated with horseradish peroxidase (Jackson Laboratories, West Grove, PA, United States) were used and immunoreactive proteins were visualized by SuperSignal West Pico chemiluminescent substrate (Thermo Scientific, Rockford, IL, United States).

### DPSC-GFP and HUASMC-mCherry cell preparation

HEK293T cells were transiently co-transfected with lentiviral packaging vectors psPAX2 and pMD2G (Vector Core, University of Michigan) with Lenti-GFP (green) or Lenti-mCherry (red) (Addgene) by the calcium phosphate method. DPSC and HUASMC were infected with lentivirus supernatants containing Lenti-GFP (green), Lenti-mCherry (red), respectively. Stable transduction of DPSC-GFP and HUASMC-mCherry was confirmed by immunofluorescence microscopy over time.

### Capillary-like sprout assay

10000 DPSC-GFP and/or HUASMC-mCherry were plated in 12-well plates coated with 550 µl/well growth factor reduced Matrigel (BD Biosciences, Bedford, MA, United States) and cultured with alpha-MEM or with vasculogenic differentiation medium (EGM2-MV + 50 ng/ml VEGF) in presence of 0–50 ng/ml rhVEGF165, 0–20 ng/ml rhbFGF and rhPDGF-BB with 0–10 µM SB203580 (Tie-2 inhibitor) or 0–25 nM Ki11502 (PDGFR inhibitor) for 7–15 days. The co-culture ratio of DPSC and HUASMC was 5 to 1. The process of capillary sprout formation was photographed over time in 6-8 random fields/well, and sprouts were counted as described (Nör et al., 1999; Mullane et al., 2008).

### SCID mouse model of vasculogenic differentiation of human dental pulp stem cells

Briefly, highly porous poly-L (lactic) acid (Boehringer Ingelheim; Ingelheim, Germany) scaffolds were seeded with 1000000 stably transduced primary human DPSC-GFP. SCID mice (CB.17. SCID; Charles River, Wilmington, MA) were anesthetized with ketamine and xylazine, and scaffolds were implanted (*n* = 2 scaffolds per mouse) in the subcutaneous space of the dorsal region of each mouse. After 3 weeks, mice (*n* = 5 per experimental condition) were treated with 5 mg/kg Ki11502 (Millipore/Sigma) by oral gavage, once a day for 7 days. At the end of the fourth week, mice were euthanized, scaffolds (*n* = 10 per experimental condition) were removed, fixed with 10% buffered formalin phosphate, and prepared for hematoxylin/eosin and immunofluorescence staining.

### Immunofluorescence and immunocytochemistry staining

Tissue slides (4 µm-thick) were deparaffinized and rehydrated. Antigen retrieval was performed with 0.5% trypsin (Sigma) at 37°C for 30–60 min. For immunofluorescence staining, slides were incubated with rabbit anti-human CD31 (Bethyl Laboratories, Montgomery, TX, United States), mouse anti-human SMA-α (Millipore/Sigma), rabbit anti-GFP (Santa Cruz Biotechnology) overnight at 4°C. Alexa Flour 488 goat anti-rabbit IgG (green) (Life Technologies) and Alexa Fluor 594 goat–anti mouse IgG (red) (Life Technologies) were used as secondary antibody. Isotype-matched non-specific IgG was used as negative control. For immunocytochemistry staining DPSC were seeded in Lab-Tek II chamber slide (Thermo/Fisher) and cultured with α-MEM supplemented with 5% FBS with or without 20 ng/ml PDGF-BB for 9 days. HDMEC were used as positive control. Slides were incubated with rabbit anti-human VE-cadherin (Cell Signaling), rabbit anti-human Occludin (Cell Signaling) overnight at 4°C, detected with MACH 3 HRP-polymer system and stained with DAB (Biocare Medical).

### Statistical analysis

One-way ANOVA followed by appropriate post-hoc tests, or t-tests, were performed using SigmaStat 4.0 software (SPSS, Chicago, IL, United States). Statistical significance was determined at *p* < 0.05.

## Results

### Vasculogenic differentiation of dental pulp stem cells *in vivo*

**FIGURE 1:**
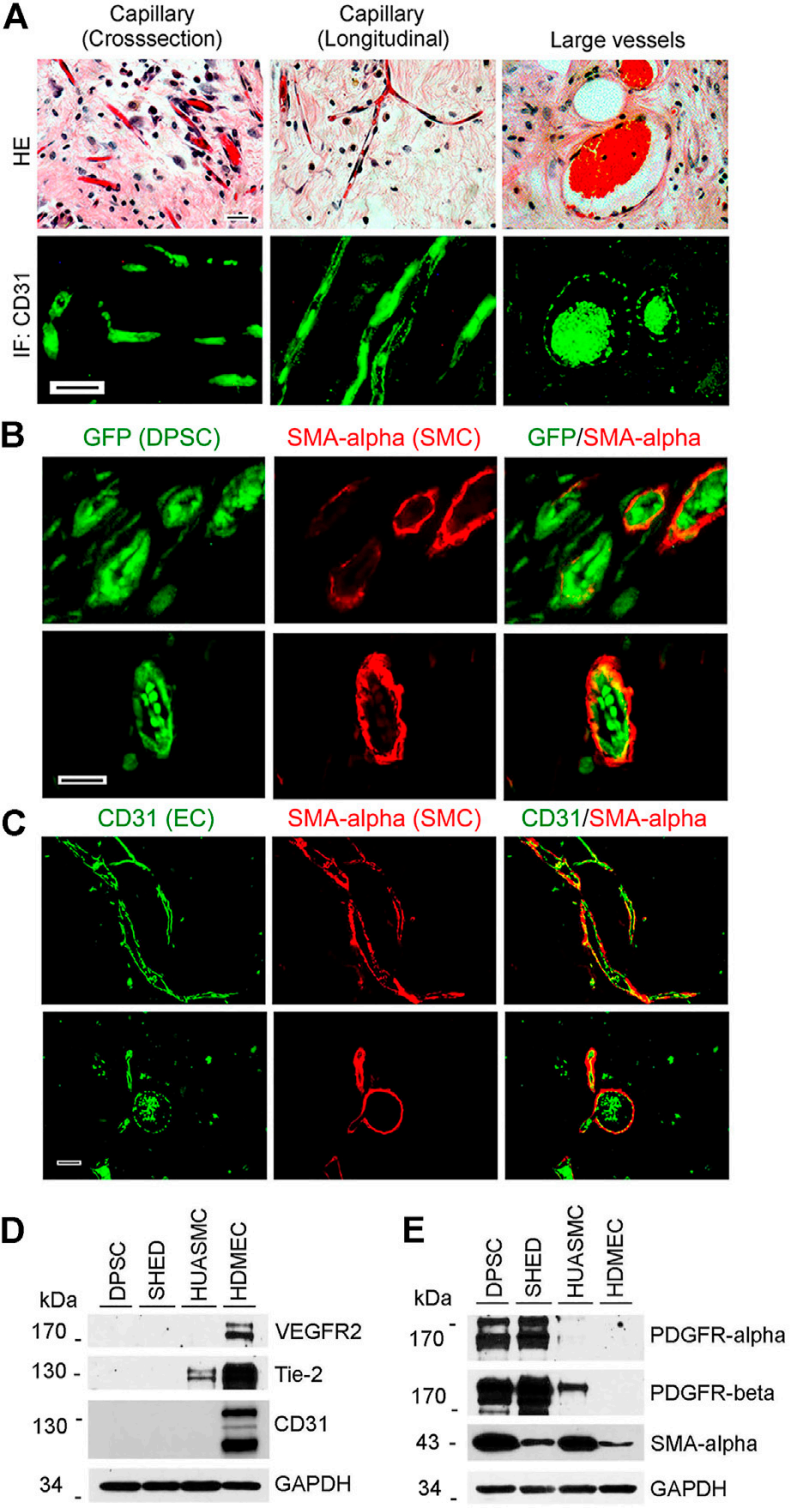
Vasculogenic differentiation of dental pulp stem cells (DPSC) *in vivo*. 1000000 GFP-transduced DPSC cells were seeded in scaffolds and transplanted into the subcutaneous space of the dorsum of SCID mice for 4 weeks. Scaffolds were retrieved and fixed with 10% buffered formaldehyde for HE and immunofluorescence staining. **(A)** HE staining showed capillaries and large blood vessels. DPSC-derived endothelial cells were positively stained with CD31. **(B)** Endothelial cells lining blood vessels were positive for GFP (i.e., generated upon vasculogenic differentiation of DPSC-GFP cells) while mural cells (pericytes/SMC) were positive for SMA-α, but not GFP (i.e., mural cells were recruited form the mouse host). **(C)** Immunofluorescence showing that endothelial cells were stained with CD31 while mural cells with SMA-α. Scale bar: 50 µm. **(D,E)** Western blots from cells (DPSC, SHED, HUASMC, and HDMEC) cultured in α-MEM supplemented with 5%FBS for the endothelial cell markers VEGFR2, Tie-2 and CD31 **(D)**, and for mural cell markers PDGFR-β and SMA-α **(E)**. HDMEC cells were used as positive control for endothelial cells, and HUASMC as controls for mural cells. GAPDH was used as loading control.

To begin to understand the process of maturation of DPSC-derived blood vessels *in vivo*, GFP transduced DPSC cells were seeded in highly porous biodegradable scaffolds and transplanted into immunodeficient mice. Histological evaluation of these scaffolds revealed presence of many capillaries in connective tissue and larger blood vessels congested with red blood cells. These vessels were positively stained by immunofluorescence (IF) with anti-human CD31 (Figure 1A). To verify that these blood vessels are derived from the transplanted human cells, DPSC stably transduced with GFP were used for transplantation (Figure 1B). Double staining for GFP and SMA-α (Figure 1B) or human CD31 and SMA-α (Figure 1C) showed that while the endothelial cells lining the blood vessels were derived from human DPSCs, the mural cells investing these blood vessels were recruited mainly from the murine host (Figures 1B,C). Of note, the spontaneous autofluorescence of red blood cells can be seen in these images and demonstrates that the DPSC-derived vessels anastomize with the host vasculature to become fully functional (blood carrying) vessels, as we showed (Sasaki et al., 2020).

To evaluate baseline expression of endothelial and mural cell markers in dental pulp stem cells from permanent teeth (DPSC), stem cells from human exfoliated deciduous teeth (SHED), human umbilical artery smooth muscle cells (HUASMC), and human dermal microvascular endothelial cells (HDMEC) we performed western blots (Figures 1D,E). We observed that unstimulated DPSC and SHED did not express endothelial markers VEGFR2, CD31, and Tie-2 at baseline. In contrast, these cells expressed PDGFR-α and PDGFR-β (Figure 1E). As such, these cells are capable of responding to PDGF stimulation.

### Maturation of DPSC-derived vascular networks *in vitro*

**FIGURE 2:**
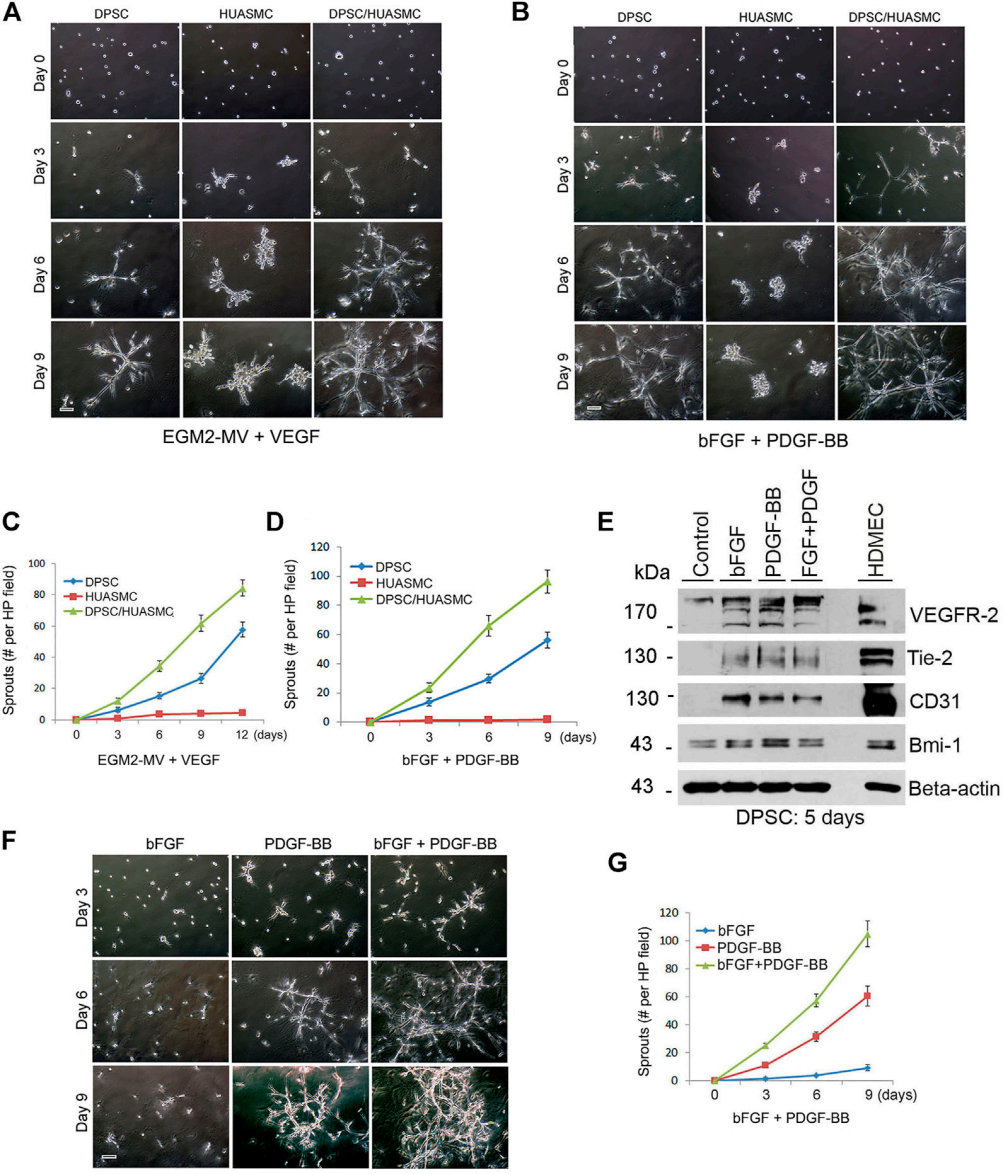
PDGF-BB and bFGF induce capillary sprouting of DPSCs. **(A–D)** 10000 DPSC or HUASMC were seeded in 12 well plates coated with growth factor reduced Matrigel and cultured with either endothelial differentiation medium-2 for microvascular cells (EGM2-MV) + 50 ng/ml VEGF as a positive controls **(A)**, or α-MEM supplemented with 5% FBS +20 ng/ml bFGF +20 ng/ml PDGF **(B)** for indicated time points. Sprouting formation was photographed and the number of sprout-like structures was counted. **(C,D)** Graphs depicting the numbers of sprouting from **(A)** and **(B)**, respectively. Scale bar: 100 µm. **(E)** DPSC cells were cultured with α-MEM supplemented with 5% FBS in presence of 20 ng/ml bFGF, PDGF-BB or bFGF + PDGF-BB for 5 days. Western blots were performed for VEGFR2, Tie-2, CD31, Bmi-1, and GAPDH (loading control). **(F)** DPSC were plated in Matrigel-coated wells and cultured with α-MEM supplemented with 5% FBS in presence of 20 ng/ml bFGF, PDGF-BB or bFGF + PDGF-BB for indicated time points. Sprout-like structures were photographed and counted. Scale bar: 100 µm. **(G)** Graph depicting the numbers of sprout-like structures from **(F)**.

To establish an experimental *in vitro* model to evaluate the process of mural cell investment of mesenchymal stem cell-derived capillaries, we cultured primary human DPSC and/or HUASMC in wells coated with growth factor-reduced Matrigel and exposed to cells to vasculogenic differentiation medium (EGM2-MV supplemented with VEGF) (Zhang et al., 2016). We observed that DPSC by themselves formed capillary-like sprouts and that co-culture of DPSC with HUASMC further increased the number of these sprouts (Figures 2A,C). Similar results were observed when DPSC and HUASMC were co-cultured with 5% FBS-MEM in presence of PDGF-BB and bFGF (Figures 2B,D). However, we did not observe the formation of capillary-like sprouts when only HUASMC cells were cultured in presence of the vasculogenic differentiation medium (Figures 2A–D; Supplementary Figure S1A).

To understand the function of bFGF and PDGF-BB on the vasculogenic differentiation of DPSC we performed Western blots that showed that both bFGF and PDGF-BB induced expression of the endothelial markers VEGFR2, Tie-2 and CD31 (Figure 2E). However, when we performed the functional capillary sprouting assay, we observed that while PDGF-BB treatment resulted in induced capillary sprouting, bFGF treatment did not (Figures 2F,G). Nevertheless, the highest number of sprouts was observed when both PDGF-BB and bFGF were combined (Figures 2F,G). Neutralizing antibody experiments blocking PDGF-BB confirmed the significance of the inductive vasculogenic signaling mediated by PDGF-BB (Supplementary Figures S1B,C).

**FIGURE 3:**
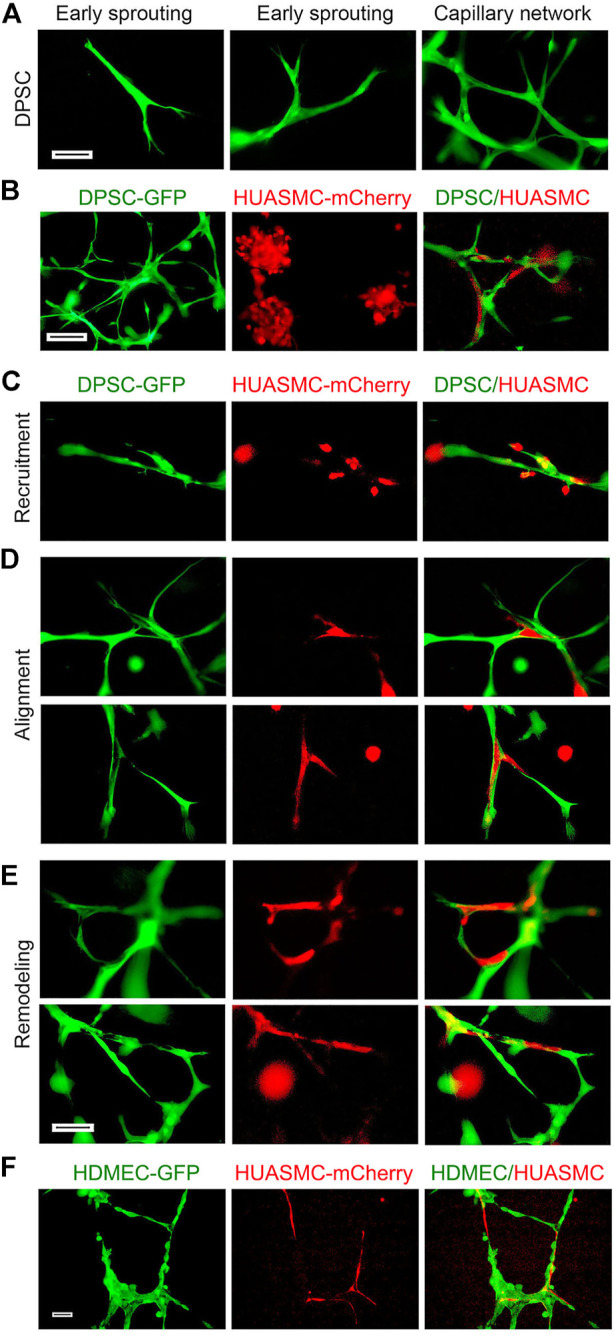
Maturation of DPSC-derived vascular networks *in vitro*. **(A–E)** 10000 DPSC (green) and HUASMC (red) were seeded in 12 well plates coated with growth factor reduced Matrigel and cultured with α-MEM supplemented with 5% FBS in presence of 20 ng/ml bFGF + PDGF-BB for 8–12 days, the sprouts were photographed with fluorescence microscope. **(A)** DPSC showed early branching, then sprouting and finally a capillary network with increasing time. **(B)** Photomicrographs of DPSC cells forming a capillary network (left); HUASMC cells that were unable to form capillary networks (middle); and HUASMC aligning with DPSC when cultured together (right). **(C)** Early stage-recruitment of HUASMC during initial spouting. **(D)** Photomicrographs showing HUASMC aligned and connecting two separate DPSC-derived branches to form initial capillary networks. **(E)** HUASMC stabilized the capillary-like structure and filled-up gaps present in the capillary networks. Scale bar: 100 µm. **(F)** 50000–100000 GFP-transduced HDMEC and mCherry-transduced HUASMC were seeded in 12 well plates coated with growth factor reduced Matrigel and cultured with EGM2-MV + 50 ng/ml VEGF for 24 h. Capillary-like networks showing HUASMC aligned with HDMEC-derived capillary-like sprouts were photographed. Scale bar: 100 µm.

Next, we explored the roles of SMC on sprouting formation and morphogenesis of dental pulp stem cells in Matrigel. Under normal physiological condition, mural cells are recruited to the blood vessels that originated from endothelial cells. Therefore, human dermal microvascular endothelial cells (HDMEC) were used as positive control (Supplementary Figure S2). Under the same culture conditions, we observed that DPSC seeded in Matrigel and exposed to vasculogenic differentiation medium (5%FBS αMEM + bFGF and PDGF-BB) exhibited typical stages of vascular morphogenesis, i.e., early branching, sprouting and eventually formation of a capillary network (Figure 3A). In contrast, HUASMC are unable to generate vascular networks by themselves, but were able to participate in DPSC-derived vascular networks when plated in co-cultures (Figure 3B). We observed that DPSC formed the initial branch and sprouting and then HUASMC were recruited to the branches (Figure 3C). Once attached to the capillary sprouts, HUASMC aligned with the DPSC-derived cells (Figure 3D), and together these cells formed complex capillary networks through remodeling (Figure 3E). As expected, HUASMC cells also aligned with endothelial cells (HDMEC) and formed complex networks (Figure 3F). Whereas DPSC and HUASMC co-cultures required at least 5 days to form vascular networks, co-cultures of endothelial cells (HDMEC) and HUASMC generated these networks in 24 h (Figure 3F; Supplementary Figure S2). We observed that during DPSC-derived vasculogenesis, HUASMC are actively recruited and play an active role in the remodeling and maturation of these vascular networks.

### Tie-2 does not contribute to the mural investment of DPSC-derived capillaries *in vitro*

**FIGURE 4:**
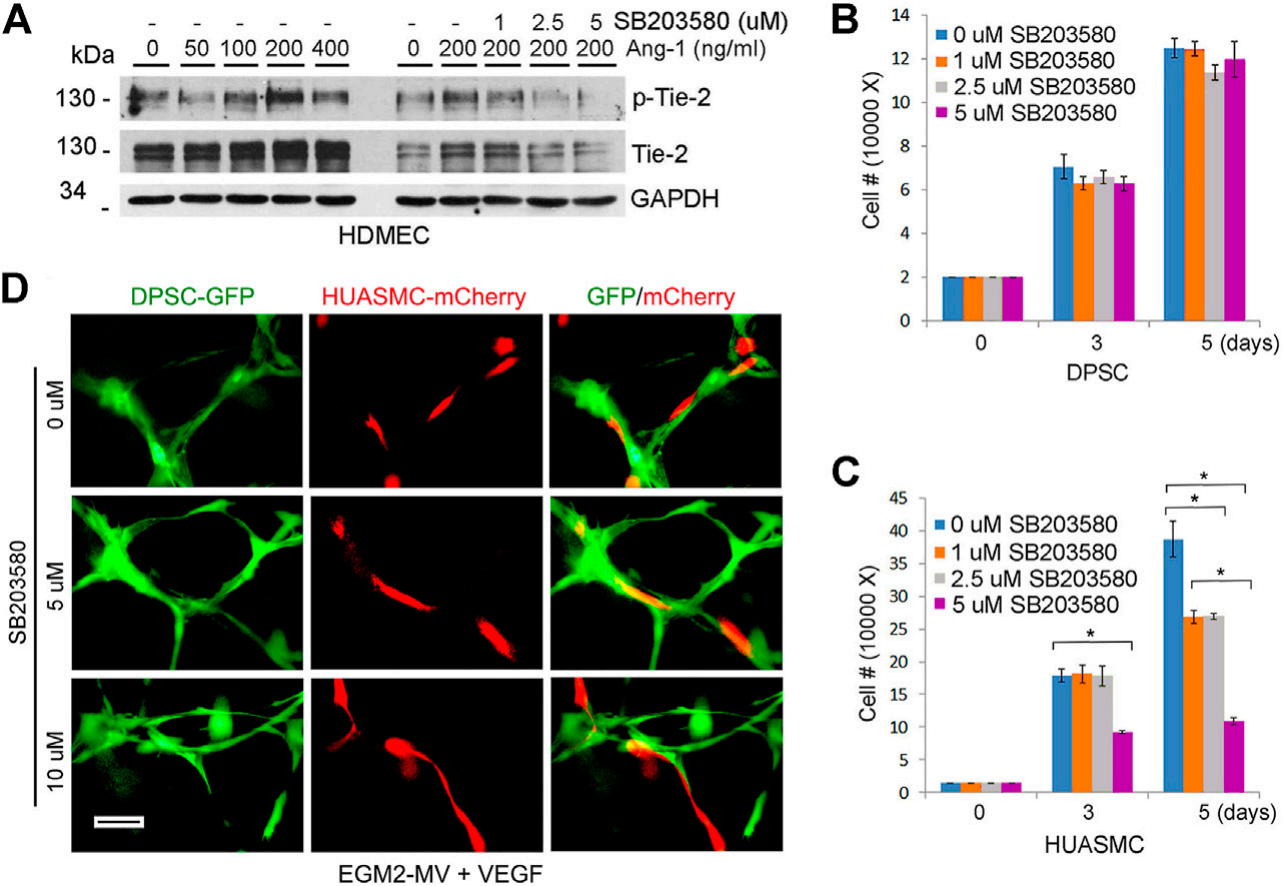
Tie-2 does not contribute to mural investment of DPSC-derived capillaries *in vitro*. **(A)** HDMEC were starved overnight and cultured with α-MEM supplemented with 5% FBS and 0–400 ng/ml Ang-1 in presence of 0–5 µM SB203580 (Tie-2 inhibitor) for 30 min. Western blots were performed for p-Tie-2, Tie-2. **(B,C)** 10000–40000 DPSC **(B)** or HUASMC **(C)** were cultured with α-MEM supplemented with 5%FBS **(B)** or SMC growth medium **(C)** in the presence of 0–5 µM SB203580. After 5 days, the number of cells were counted under microscopic evaluation. Graphs depicting the number of DPSC **(B)** and HUASMC **(C)** in response to the Tie-2 inhibitor. **(D)** 10000 DPSC or HUASMC were seeded in 12 well plates coated growth factor reduced Matrigel and cultured with EGM2-MV + 50 ng/ml VEGF in presence of 0–10 µM SB203580 for indicated time points. Microphotographs of networks composed of DPSC-derived capillary-like structures lined with HUASMC cells.

Angiopoietin/Tie (Ang/Tie) signaling regulates the association of vascular endothelial cells and pericytes in physiological settings (Augustin et al., 2009). In contrast to DPSC and SHED cells, HUASMC cells express Tie-2 (Figure 1D). Therefore, it is plausible that Tie-2 signaling plays a role on the regulation of vasculogenic differentiation and maturation of DPSC-derived capillaries. First, we verified that Ang-1 induced dose-dependent phosphorylation of Tie-2, and that SB203580 (Tie-2 kinase inhibitor) was effective at blocking Tie-2 activity (Figure 4A). Then, we exposed DPSC cells to increasing concentrations of SB203580 and observed that the Tie-2 inhibitor did not have a cytotoxic effect on these cells (Figure 4B). In contrast, this inhibitor did show a dose- and time-dependent effect on the density of HUASMC (Figure 4C). Nevertheless, treatment with SB203580 did not have a significant effect on the investment with smooth muscle cells of DPSC-derived capillaries (Figure 4D).

### PDGFR-β signaling regulates the investment and maturation of DPSC-derived capillaries

**FIGURE 5:**
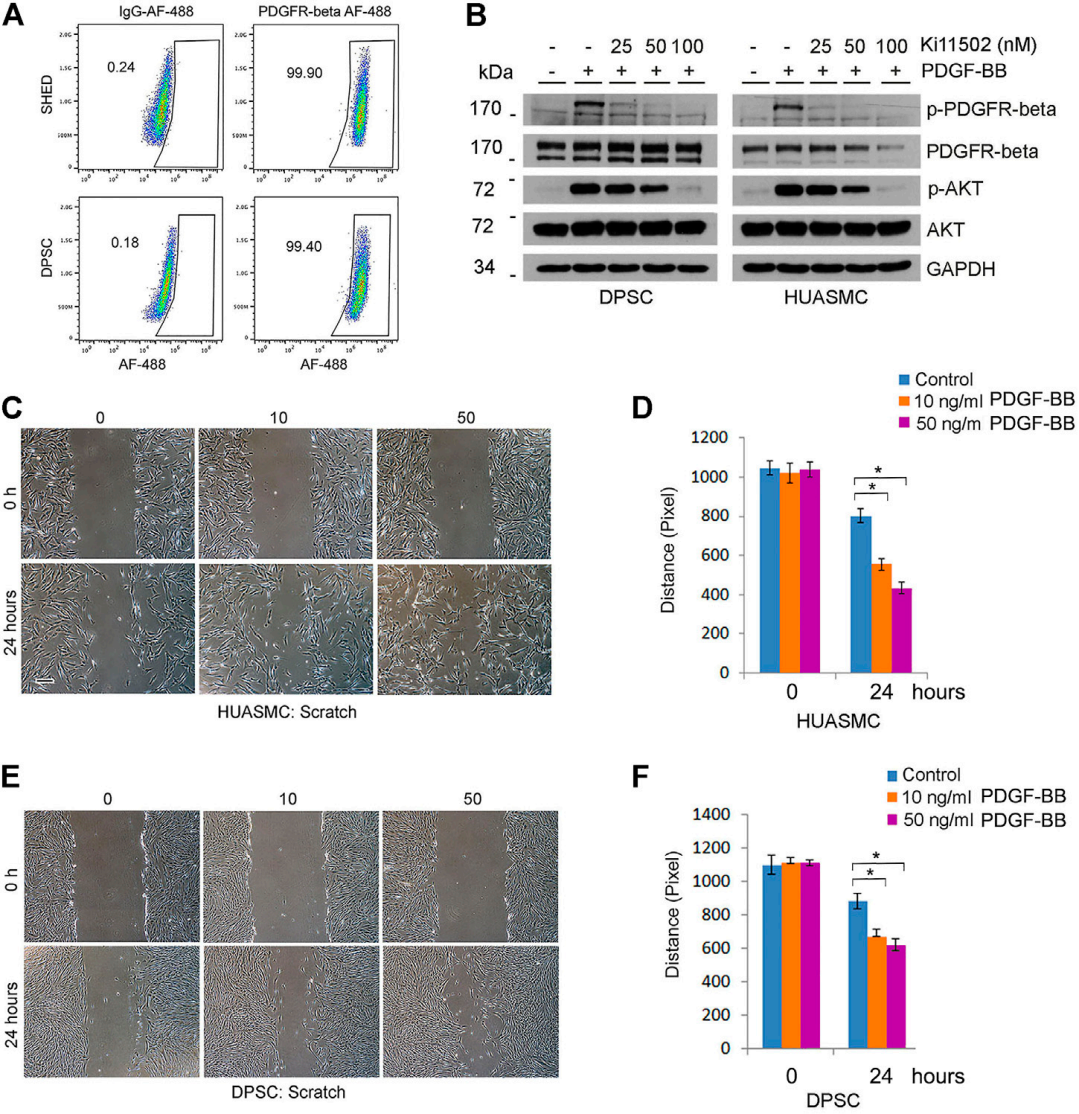
PDGFR-β signaling induces motility of DPSC cells and smooth muscle cells *in vitro*. **(A)** Flow plots depicting the expression of PDGFR-β in DPSC and SHED. IgG-AF488 was used as isotype control. **(B)** DPSC and HUASMC were starved overnight and pre-incubated with 0–100 nM Ki11502 (PDGFR inhibitor) for 2 h, and then treated with α-MEM supplemented with 5% FBS in presence of 20 ng/ml PDGF-BB for 30 min. Western blots were performed for p-PDGFR-β, PDGFR-β, p-AKT, and AKT. **(C–F)** HUASMC **(C)** and DPSC **(E)** were cultured in 6-well plates, starved overnight, scratched with a sterile 1,000 µl loading tip, then treated with α-MEM supplemented with 5% FBS in presence of 0–50 ng/ml PDGF-BB for indicated time points. Scale bar: 100 µm. **(D,F)** Graphs depicting the scratch width over time in response to PDGF-BB in HUASMC **(C)** and DPSC **(E)**. Three independent experiments using triplicate wells/experimental condition were performed to verify reproducibility of the data. Asterisk indicates *p* < 0.05.

Since Tie-2 signaling did not play a significant role on the investment of capillaries generated by DPSC, we explored an alternative pathway, i.e., PDGF-BB signaling through PDGFR-β (Lindblom et al., 2003). Flow cytometry analysis showed that nearly 100% DPSC and SHED are positive for PDGFR-β (Figure 5A). As expected, treatment with recombinant human PDGF-BB induced phosphorylation of PDGFR-β and downstream AKT in both DPSC and HUASMC cells (Figure 5B) but did not enhance cell proliferation (Supplementary Figures S3A,B). To confirm specificity of these responses, we used Ki11502, a novel receptor tyrosine kinase (RTK) inhibitor of PDGFR-β phosphorylation and proteoglycan synthesis (Nishioka et al., 2008; Getachew et al., 2010). We observed that Ki11502 inhibited PDGFR-β and AKT phosphorylation in both cell types (Figure 5B).

**FIGURE 6:**
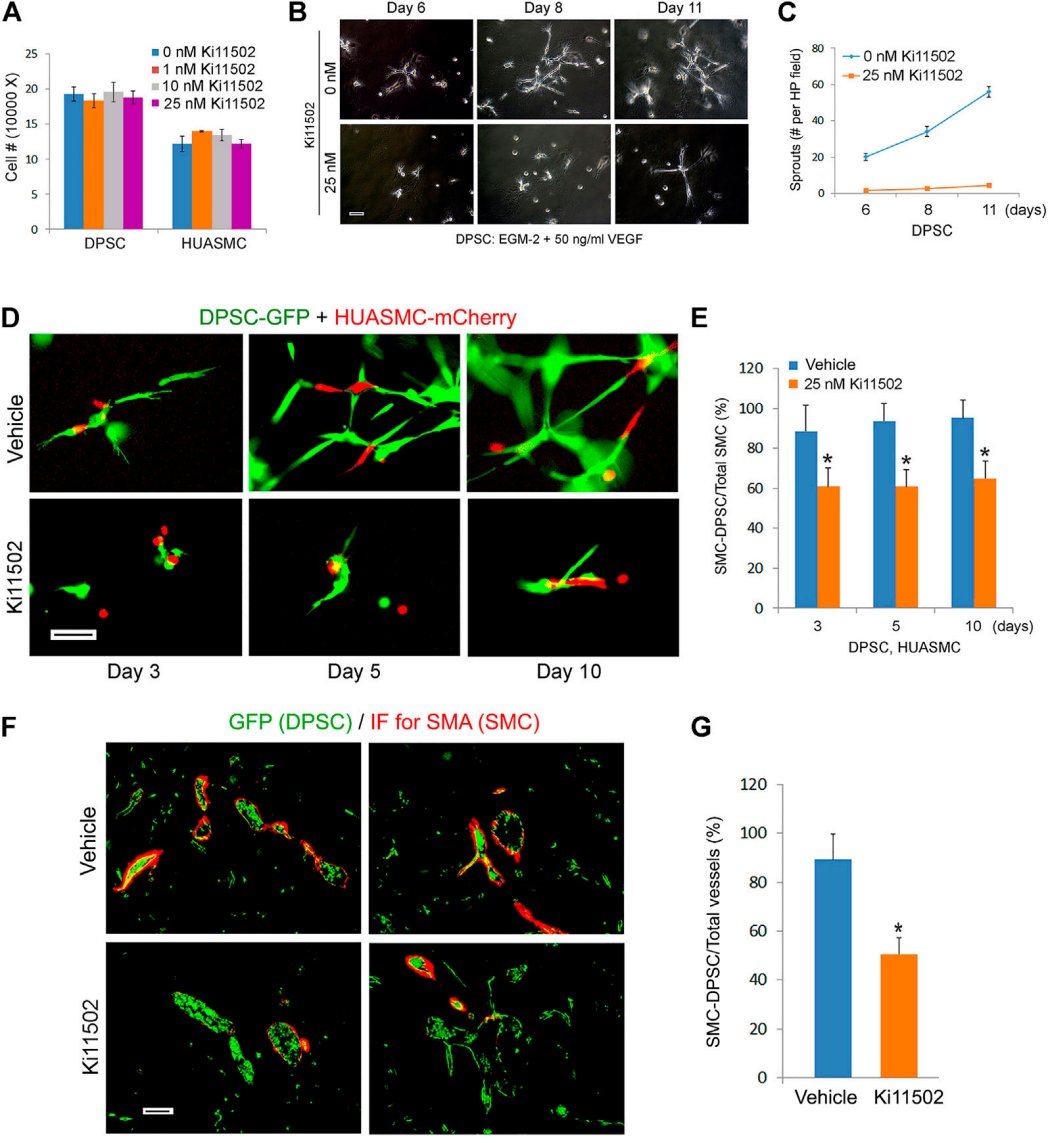
Blockade of PDGFR-β signaling inhibits maturation of DPSC-derived blood vessels *in vitro* and *in vivo*. **(A)** 10000–50000 DPSC or HUASMC were cultured with α-MEM supplemented with 5% FBS in presence of 0–25 nM Ki11502. After 4 days, the number of cells were counted under microscopic evaluation. **(B,C)** 10000 DPSC were plated in growth factor-reduced Matrigel-coated wells and cultured with EGM2-MV + 50 ng/ml VEGF in presence of 0 or 25 nM Ki11502 (PDGFR inhibitor) for indicated time points. Photomicrographs of capillary-like sprouts. Scale bar: 100 µm. **(C)** Graph depicting the number of capillary-like sprouts from **(B)**. **(D,E)** DPSC (green) and HUASMC (red) were seeded in Matrigel-coated plates and cultured with α-MEM supplemented with 5% FBS in presence of 20 ng/ml bFGF and PDGF-BB in the presence of 25 nM Ki11502 for indicated time points. Vascular networks were photographed. Scale bar: 100 µm. **(E)** Graph depicting the percentage of HUASMC cells in direct contact with DPSC cells as a fraction of the total number of HUASMC cells in the microscopic field. Asterisks depict *p* < 0.001. **(F,G)** 1000000 GFP-transduced DPSC cells were seeded in scaffolds and transplanted into the subcutaneous space of immunodeficient mice. After 3 weeks, mice (*n* = 5 per group) were treated with vehicle (control) or with 5 mg/kg Ki11502 by oral gavage, once a day for 7 days. After 4 weeks, mice were euthanized, scaffolds were removed, fixed, and prepared for HE and immunofluorescence staining. **(F)** Immunofluorescence for GFP (DPSC, green) and SMA-α (mouse mural cells, red) double staining. **(G)** Graph depicting the percentage of blood vessels covered by SMA-α cells as compared to total blood vessels from **(F)**. Asterisk depicts *p* < 0.001.

To begin to understand the function of PDGF-BB on investment of DPSC-derived capillaries, we performed a scratch assay that showed that PDGF-BB increased the motility of both DPSC and HUASMC (Figures 5C–F). Then, we performed *in vitro* capillary sprout assays using the minimum concentration of Ki11502 (25 nM) that effectively inhibited PDGFR-β phosphorylation (Figure 5B) without affecting DPSC or HUASMC viability/proliferation (Figure 6A). Under these experimental conditions, we observed a significant decrease in DPSC-derived capillary-like sprouts (Figures 6B,C). Importantly, when we co-cultured DPSC-GFP and HUASMC-mCherry in presence of the vasculogenic differentiation medium supplemented with 25 nM Ki11502 (Figure 6D), we observed a significant decrease in the proportion of mCherry-positive cells investing GFP-positive capillary-like structures at all time points evaluated (Figure 6E).

Adherens junctions are cell–cell adhesion complexes that make important contributions to development and tissue homeostasis (Harris and Nelson., 2010). VE-cadherin is an endothelial specific adhesion protein located at junctions between endothelial cells (Vestweber, 2008). To evaluate the presence of intercellular junctions in dental pulp stem cells that have undergone vasculogenic differentiation, DPSC were cultured in presence of 0 or 20 ng/ml PDGF-BB for 9 days. Immunocytochemistry showed that PFGF-BB-treated DPSC cells were positively stained with VE-Cadherin in the cytoplasm and cell-cell contact areas, while untreated cells did not stain for VE-cadherin (Supplementary Figure S4A). In contrast, DPSC treated with PDGF-BB did not stain for Occludin *in vitro* (Supplementary Figure S4A). To verify these results, we also performed immunohistochemistry for Occludin of a DPSC-derived blood vessel and of a human dental pulp. In both cases, the blood vessels were not stained for Occludin. As a positive control for Occludin immunohistochemistry, we used a tissue section from a xenograft head and neck squamous cell carcinoma generated with UM-SCC-1 cells that shows clear staining for Occludin at the cell membrane (Supplementary Figure S4B).

To confirm and validate *in vitro* results, we performed an *in vivo* study with primary human DPSC-GFP cells seeded in highly porous biodegradable scaffolds and transplanted into the subcutaneous space of immunodeficient mice. Three weeks after transplantation, Ki11502 was delivered by oral gavage, once a day for 7 days. The day after the last dose of Ki11502, the mice were euthanized, scaffolds were retrieved and processed for histology. We observed that the endothelial cells lining blood vessels inside the scaffolds were positive for GFP, which demonstrates that they were formed by the human DPSCs that were transplanted in the mice (Figure 6F). Since human DPSCs express SMA-α (Figure 1E), some of these blood vessels are depicted in yellow (Figure 6F). In contrast, the smooth muscle cells investing these DPSC-derived blood vessels stained positive for SMA-α but were negative for GFP as demonstrated by their red color only (Figure 6F) indicating that these cells have been recruited from the host. Importantly, inhibition of PDGFR-β signaling with Ki11502 reduced the investment of GFP-positive vessels with SMA-α positive cells (*p* < 0.05), when compared to vehicle controls (Figures 6F,G).

## Discussion

The dental pulp tissue has been characterized as a rich source of mesenchymal stem cells (MSC). These cells express not only MSC markers (e.g., CD44, CD90, CD105, CD73) but also VEGFR1 that enables these cells to respond to vasculogenic stimuli (Bento et al., 2013; Sakai et al., 2010; Zhang et al., 2016; Zhang et al., 2021). Here, we also showed that these cells express PDGFR-β enabling them to respond to signaling triggered by PDGF-BB, a major regulator of the process of mural cell investment of blood vessels. Mural cells are necessary to stabilize vessels through physical and molecular interactions with adjacent endothelial cells. As such, absence of mural cells may lead to vascular leakage and hemorrhaging (Armulik et al., 2010; Armulik et al., 2011; Daneman et al., 2010). In previous studies, we have demonstrated that DPSC cells can differentiate into functional blood vessels when seeded in polylactic acid scaffolds and transplanted in the subcutaneous space of immunodeficient mice (Sakai et al., 2010; Sasaki et al., 2020; Zhang et al., 2016; Zhang et al., 2021; Mantesso et al., 2021). These polylactic acid scaffolds are biodegradable, highly porous, and provide adequate conditions for the vasculogenic differentiation of dental pulp stem cells (Sakai et al., 2010; Sakai et al., 2011). We have observed that many DPSC-generated vessels were invested by mural cells. However, the origin and the mechanisms for recruitment of these cells to the DPSC-generated blood vessels was unclear. Here, we showed that the host mainly contributes with mural cells investing these blood vessels and that PDGF-BB signaling through PDGFR-β plays a major role in the process of recruitment of these mural cells from the host.

We used two kinds of culture medium to study vasculogenic morphogenesis, as follows: 1) EGM2-MV and 50 ng/ml VEGF; or 2) α-MEM 5% FBS with 20 ng/ml bFGF, and 20 ng/ml PDGF-BB. VEGF synergizes with basic fibroblast growth factor (bFGF) to induce pro-angiogenic responses, while PDGF-BB has a critical role in the stabilization of newly formed physiological blood vessels (Senger et al., 1983; Shing et al., 1984; Yancopoulos et al., 2000; Ferrara, 2002). Angiogenic endothelial cells secrete PDGF-B and recruit PDGFR-β expressing pericytes (Bjarnegard et al., 2004). Combination of PDGF-BB and bFGF markedly stimulate arteriogenesis with a significant increase in vascularization and improvement in blood flow (Cao et al., 2003). In disease, bFGF and PDGF-BB synergistically promote murine tumor neovascularization and metastasis (Nissen et al., 2007). These colleagues showed that bFGF acts as a sensitizer for endothelial cells to respond to PDGF-BB, which feeds back to vascular smooth muscle cells to enhance their responses to bFGF stimulation (Nissen et al., 2007). We observed that bFGF alone cannot induce vascular sprouting. However, when combined with PDGF-BB, bFGF can increase the number of capillary-like sprouts. A possible explanation for these results is that while bFGF does not have a vascular morphogenesis effect by itself, it is a potent inducer of endothelial cell proliferation and differentiation. As such, once sprouting is initiated by bona fide pro-angiogenic factors (e.g., VEGF), bFGF can augment the vascularization by inducing endothelial cell proliferation and differentiation.

We have also observed that, while HUASMC cells were unable to generate capillary-like sprouts under our experimental conditions. However, when co-cultured with DPSC, we observed increased numbers of capillary-like sprouts when compared with single DPSC cultures. To understand *in vitro* vasculogenesis mediated by co-cultures of DPSC and HUASMC, we used GFP-transduced human dental pulp stem cells and mCherry-transduced human smooth muscle cells. As positive controls, we used GFP-transduced primary human endothelial cells co-cultured with mCherry-transduced human smooth muscle cells. Since HDMEC are fully differentiated mature cells, capillary-like sprout formation was already observed within 24 h, as we have shown (Zhang et al., 2010). Under the same conditions, DPSC also form capillary networks but it takes longer, i.e., at least 5 days. This delay in vascular network formation is likely due to the fact that it takes about 5–7 days for DPSC cells to differentiate into endothelial cells (Sasaki et al., 2020). This might also explain the fact that we do not observe significant degradation of the Matrigel during this time period, as we will only have fully differentiated endothelial cells capable of degrading the matrix in the last few days of the experimental period.

When DPSC are co-cultured with HUASMC, the DPSCs make the initial branches and then HUASMC cells are recruited to the branch and aligned with it, stabilizing the growing vascular networks. The alignment and binding of both cell types happens with such a display of cellular organization that is unlikely to be achievable through random events and that certainly required a functional crosstalk between the 2 cell types that is mediated by soluble factors and by intimate cell-cell interactions (e.g., intercellular junctions). Indeed, we observed that this process is regulated by PDGF-BB signaling through PDGFR-β, not *via* angiopoietin signaling through Tie-2, which was our original hypothesis. After extensive studies with inhibitors of Tie-2 signaling, we concluded that while this is a critical pathway for the physiological investment of blood vessels generated by endothelial cells, it does not seem to play a major role in the investment of blood vessels generated by DPSC cells. The possible reasons for this difference are beyond the scope of this work but are going to be investigated in the laboratory as a continuation of this project.

We used several strategies to understand the function of PDGF-BB signaling through PDGFR-β in the regulation of the investment of DPSC-generated blood vessels by smooth muscle cells. We observed that PDGF-BB induced phosphorylation of PDGFR-β and downstream AKT (major regulator of cell survival and proliferation) in both DPSC and HUASMC and enhanced vascular morphogenesis. Blockade of PDGFR-β signaling pathway inhibited PDGF-BB-induced AKT phosphorylation and capillary-like sprout formation. Importantly, inhibition of PDGFR-β compromised smooth muscle cell recruitment, association, and alignment with DPSC-generated capillary-like sprouts *in vitro*. *In vivo*, we observed that mural cells start to cover DPSC-derived blood vessels on the third week after DPSC transplanted into mice. As such, we started treating the mice systemically with the PDGFR-β inhibitor 21 days after transplantation and continued treatment for 1 week. We observed that inhibition of PDGFR-β signaling reduced the association of SMA-positive mural cells to DPSC-derived blood vessels, confirming our *in vitro* results.

DPSCs have been considered ideal stem cell sources for tissue engineering. Some research groups have shown that when co-cultured with human endothelial cells, DPSCs function as pericytes that stabilize capillary-like structures and promote vascular maturation (Janebodin et al., 2013; Monache et al., 2019). Here, we leveraged the vasculogenic differentiation capacity of DPSCs to vascularize the tissue and found that host smooth muscle cells can be recruited to invest DPSC-derived vessels. While our work does not contradict previous findings that DPSCs can function as pericytes, it suggests that one does not have to pre-vascularize scaffolds or co-transplant endothelial cells to engineer highly vascular and viable tissues with DPSCs. However, scaffold size and cell numbers associated to it may influence cell’s requirements prior to transplantation.

In summary, our data demonstrated that DPSC can differentiate into vascular endothelial cells that form functional blood vessels that are matured upon recruitment and investment of host smooth muscle cells. The process of DPSC-derived vessel maturation is regulated by PDGF-BB signaling through PDGFR-β. This is a critical cellular event that is required for the stabilization and long-term function of blood vessels. As such, the work presented here provides insights that can be used for the development of mechanism-based therapies for tissue regeneration that involve vasculogenic differentiation of mesenchymal stem cells.

## Data Availability

The raw data supporting the conclusion of this article will be made available by the authors, without undue reservation.
