# Supplementary material for: PDGF-BB signaling via PDGFR-β regulates the maturation of blood vessels generated upon vasculogenic differentiation of dental pulp stem cells
Source: Front Cell Dev Biol. 2022 Oct 19;10:977725. doi: 10.3389/fcell.2022.977725 (PMC9627550; doi:10.3389/fcell.2022.977725)
Supplement: Supplementary file 1 [file DataSheet1.PDF]

Supplementary Figure Legends

Supplementary Fig. S1: Inhibition of PDGF-BB blocks DPSC-derived capillary sprouting. **(A)** $1X10^4$ HUASMC were grown in growth factor reduced Matrigel and cultured with 5% FBS a-MEM in presence of 20 ng/ml bFGF, PDGF-BB, and bFGF + PDGF-BB, respectively, for indicated time points. All the stimuli did not induce sprouting formation in HUASMC. Scale bar: 100 µm. **(B, C)** $1 X 10^4$ DPSC and HUASMC were grown in Matrigel and cultured with 5%FBS a-MEM + 20 ng/ml bFGF and PDGF-BB, with or without 0.6 ug/ml PDGF-BB neutralizing antibody for indicated time points, sprouts were photographed. Scale bar: 100 µm. **(C)** graph depicting the numbers of sprouting in **(B)**.

Supplementary Fig. S2: Capillary sprout formation by primary human endothelial cells (HDMEC). **(A, B)** 5-10 X 10^4 HDMEC and HUASMC were seeded in growth factor reduced Matrigel coated 12 well plate and cultured with EGM-2 for 24 h. Tube-like structures were photographed in HDMEC, HDMEC and HUASMC **(A),** and GFP transduced HDMEC **(B)**. Scale bar: 50 µm.

Supplementary Fig. S3: PDGF-BB induces AKT signaling, but not proliferation, of DPSCs. **(A)** DPSC and SHED were starved overnight and stimulated with 0-20 ng/ml PDGF-BB for 20 min, western blots were performed for p-AKT, AKT. **(B)** 5-10 X 10^3 SHED and DPSC were cultured with regular medium in 12 well plate in the presence of 0-20 ng/ml PDGF-BB for 3 days, the cells were collected and counted. The graphs represented one of three independent experiments.

Supplementary Fig. S4: PDGF-BB induces VE-cadherin but not Occludin in DPSC cells. **(A)** DPSC cells were seeded in chamber slides and cultured 0 or 20 ng/ml PDGF-BB for 9 days. Immunocytochemistry staining for VE-cadherin and for Occludin. **(B)** Immunohistochemistry for Occludin in DPSC-derived blood vessels and blood vessels from a human dental pulp. As positive control for Occludin, we performed IHC of a xenograft human head and neck squamous cell carcinoma generated with UM-SCC-1 cells. Scale bars represent 25 µm.

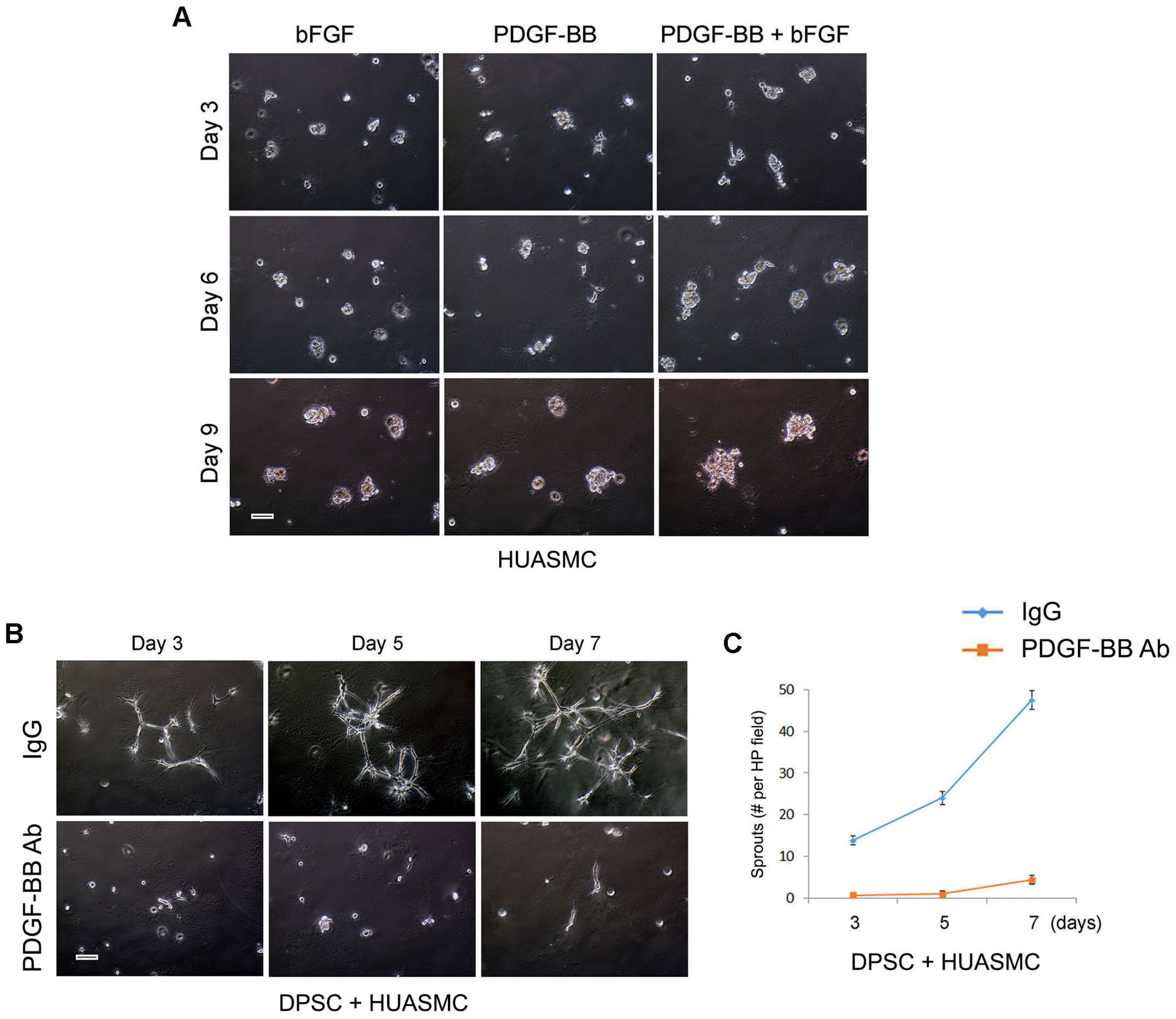

Supplementary Fig. S1 Zhang et al

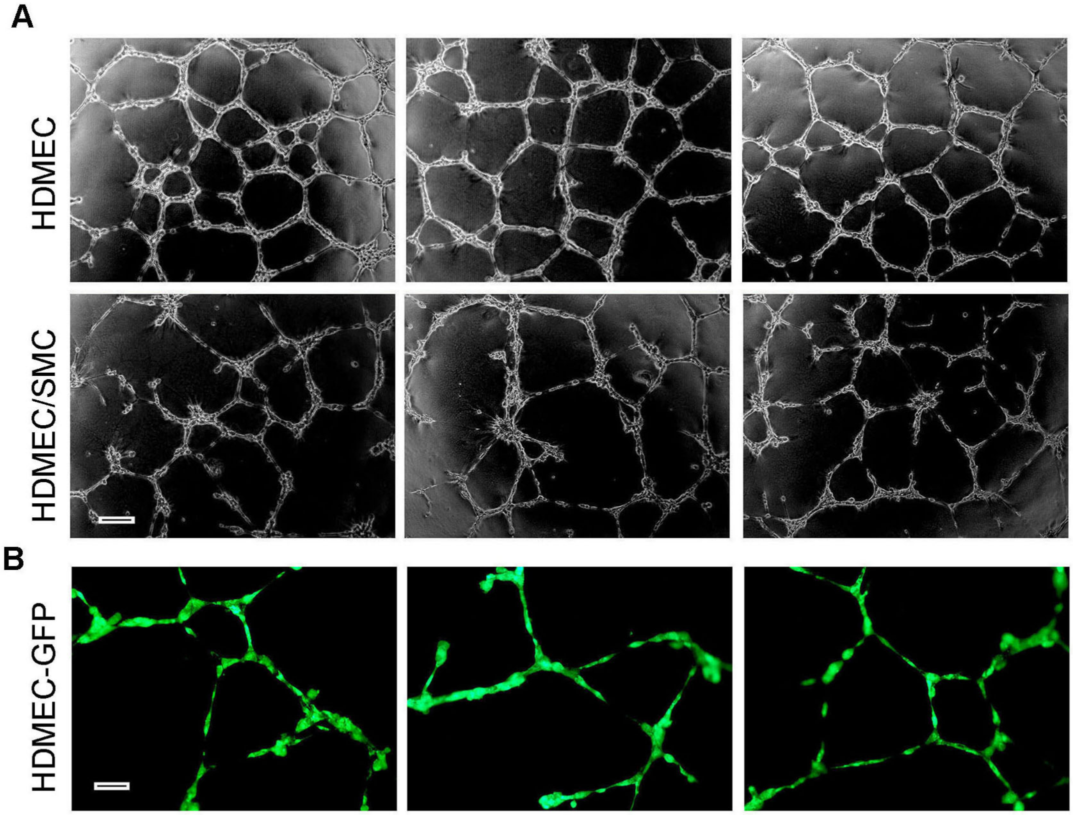

Supplementary Fig. S2 Zhang et al

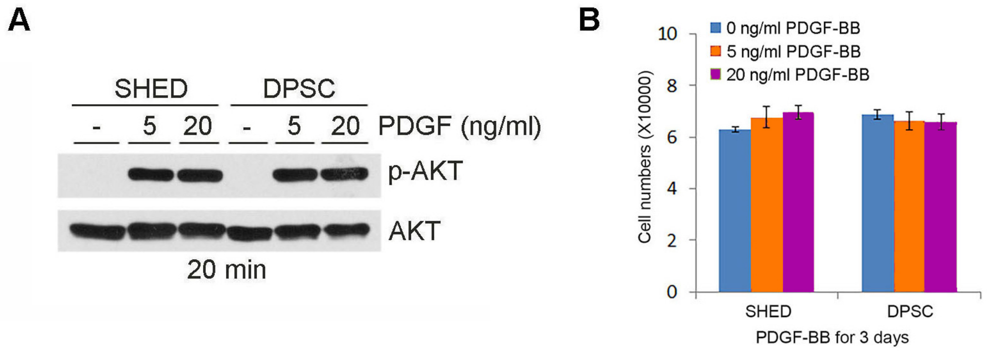

Supplementary Fig. S3 Zhang et al

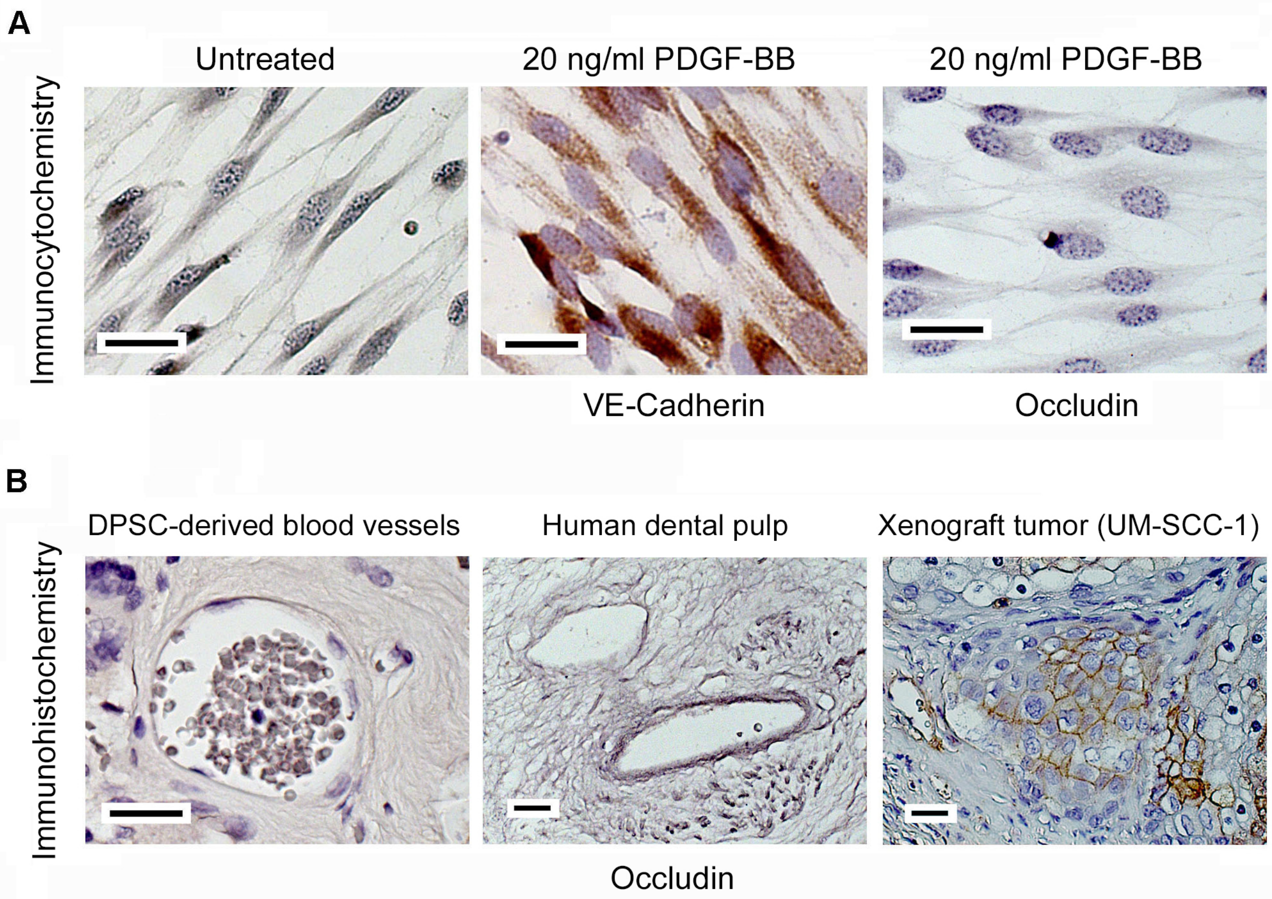

Supplementary Figure S4 - Zhang et al
